# Amplification of Duffy binding protein-encoding gene allows *Plasmodium vivax* to evade host anti-DBP humoral immunity

**DOI:** 10.1038/s41467-020-14574-9

**Published:** 2020-02-19

**Authors:** Jean Popovici, Camille Roesch, Lenore L. Carias, Nimol Khim, Saorin Kim, Amelie Vantaux, Ivo Mueller, Chetan E. Chitnis, Christopher L. King, Benoit Witkowski

**Affiliations:** 1 Malaria Molecular Epidemiology Unit, Malaria Translational Research Unit, Institut Pasteur du Cambodge, Institut Pasteur, Phnom Penh, Cambodia; 2 Center for Global Health and Diseases, Case Western Reserve University, School of Medicine, Cleveland, OH 44106 USA; 3 Department of Parasites and Insect Vectors, Institut Pasteur, Paris, France; 4 Population Health and Immunity Division, The Walter and Eliza Hall Institute of Medical Research, Parkville, VIC Australia; 5 Department of Medical Biology, The University of Melbourne, Parkville, VIC, Australia; 6 Cleveland VA Medical Center, Cleveland, OH 44106 USA

**Keywords:** Antibodies, Parasite immune evasion, Pathogens, Malaria

## Abstract

Antigenic variation, the capacity to produce a range of variable antigens, is a well-described strategy of *Plasmodium* and other parasites to evade host immunity. Here, we show that gene amplification is an additional evasion mechanism used by *Plasmodium vivax* to escape humoral immunity targeting PvDBP, the key ligand involved in reticulocyte invasion. PvDBP gene amplification leads to increased mRNA levels and protects *P. vivax* in vitro against invasion inhibitory human monoclonal antibodies targeting a conserved binding domain of DBP. Patient samples suggest that parasites with increased *pvdbp* copy number are able to infect individuals with naturally acquired antibodies highly blocking the binding of PvDBP to the Duffy receptor. These results show that gene copy number variation affect the parasite’s ability to evade anti-PvDBP humoral immunity.

## Abstract (web summary)

Duffy binding protein (DBP) of *Plasmodium vivax* is important for invasion and is a potential vaccine candidate. Here, the authors show that PvDBP gene amplification protects *P vivax* in vitro against invasion inhibitory human monoclonal antibodies and is associated to infection of patients with PvDBP binding inhibitory antibodies.

## Introduction

Intracellular parasite proteins involved in receptor-ligand interactions mediating the invasion of host cells are under strong immune pressure. A fine balance must be maintained to ensure on the one hand escape from host immunity and on the other, conservation of ligand domains interacting with host cell receptors. In *Plasmodium vivax* (Pv), the geographically most widespread human malaria parasite^1^, the Pv Duffy-Binding Protein (PvDBP) is a typical example of this fine balance with polymorphic antigenic domains and conserved residues within the protein region (region II, DBPII) binding to the host cell receptor, the Duffy antigen (also known as Duffy Antigen Receptor for Chemokine, DARC). The PvDBP-Duffy interaction is critical for the invasion of the parasite into reticulocytes, the target host cell for Pv, as individuals lacking the Duffy antigen on their erythrocytes (Duffy-negative) are resistant or have markedly reduced susceptibility to Pv infection^2,3^. PvDBP is thus a leading candidate for a blood-stage vaccine against this parasite^2,4–8^. While there is significant polymorphism in DBPII, the binding residues are conserved^9–15^. Although individuals residing in Pv endemic areas commonly have antibodies to DBPII non-binding immuno-dominant residues, only a minority of patients can develop antibodies targeting binding amino acids of the protein resulting in strain-transcending naturally acquired immunity against Pv^16–20^. Yet some individuals with high titers of these naturally acquired antibodies remain susceptible to malaria infection and disease suggesting alternative mechanisms to antigenic diversity that the parasite might have evolved to escape this strain-transcending immunity. Recently it was shown that some Pv parasites had two or more copies of the gene coding for PvDBP^21–23^. Such Pv isolates were identified from many endemic areas around the world. Because of the observation that Pv could occasionally invade Duffy negative cells^24,25^, it was hypothesized that the role for this gene amplification was to facilitate binding to an alternative lower affinity receptor in Duffy negative reticulocytes^21,22,25^. However, such an alternative receptor has not been identified and Pv with multiple copies are frequently observed in Pv endemic population where Duffy negative individuals are rare. Here we show that PvDBP gene amplification allows Pv to evade host anti-PvDBP humoral immunity.

## Results

### *pvdbp* amplification leads to increased mRNA

**Fig. 1:**
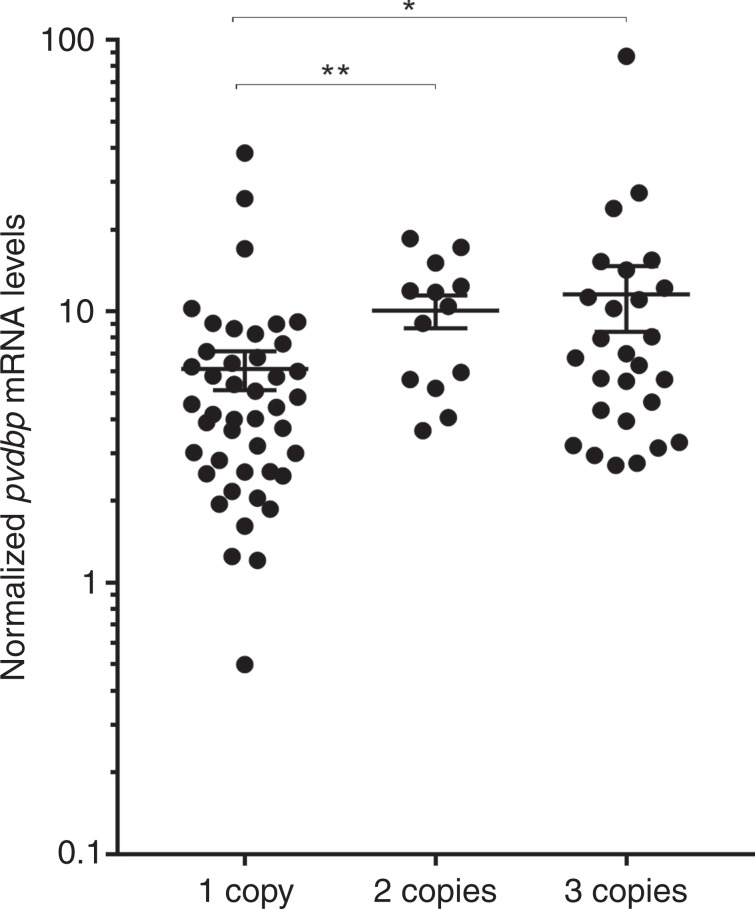
Increased PvDBP gene copy number leads to increased mRNA levels in mature Pv schizonts. PvDBP mRNAs were normalized against the schizont-specific PvMSP1 gene. mRNA level is on average higher in parasites with two (*n* = 13) and three (*n* = 27) copies of *pvdbp* compared to single copy (*n* = 44) ones (Kruskal-Wallis H = 12.39, *P* = 0.020, Dunn’s post-hoc tests, *P < 0.05, **P < 0.001). Each dot represents the *pvdbp* mRNA quantification of a different clinical isolate. Mean ± SEM. Source data are provided as a Source Data file.

To determine if *pvdbp* amplification was associated with higher gene transcription, we evaluated mRNA levels in parasites matured in vitro until a majority reaches the schizont stage when merozoites are fully developed. *pvdbp* mRNA was normalized against mRNA of the PvMSP1 gene which is single copy and also expressed at the schizont stage^26,27^. On average, parasites with a single *pvdbp* copy had a lower *pvdbp* mRNA level (mean = 6.14 arbitrary units ± 1.01 SEM) compared to parasites with two (mean = 10.07 ± 1.39 SEM) and three copies (mean = 11.56 ± 3.15 SEM) (Dunn’s post-hoc tests *P* = 0.0045 and *P* = 0.0203 respectively) (Fig. 1a). We attempted to determine if increased *pvdbp* copy number leads to increased protein levels by flow cytometry using polyclonal anti-PvDBP (Supplementary Methods). However, variance between samples was high and didn’t allow conclusive results on PvDBP protein expression, particularly given the low number of available samples (Supplementary Fig. 1).

### *pvdbp* amplification protects Pv in vitro against antibodies

**Fig. 2:**
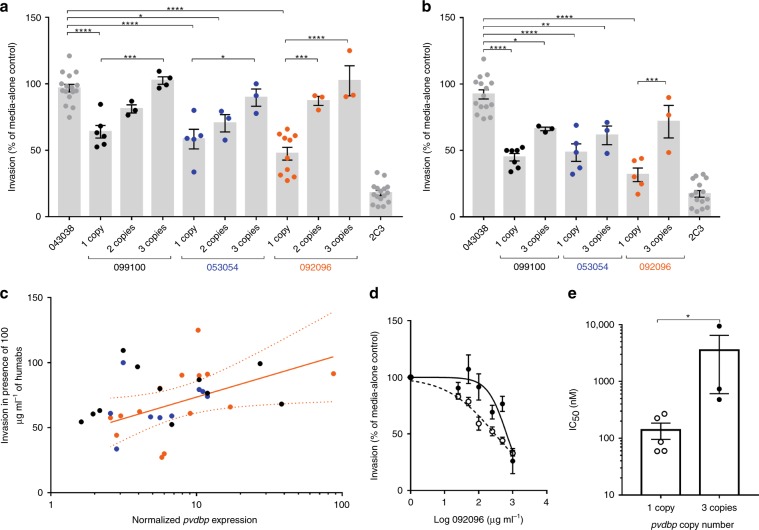
Increased PvDBP gene copy number protects Pv against in vitro reticulocytes’ invasion inhibition activity of human monoclonal anti-PvDBP antibodies. **a** Invasion by parasites with 1, 2 or 3 copies of *pvdbp* in presence of 100 μg ml^−1^ of the humabs 099100, 053054 and 092096 is normalized to media-alone controls. Negative control is made of the isotype-matched humab 043038 and the positive control is made of the anti-Duffy monoclonal antibody 2C3. Each dot represents the average inhibition of a single clinical isolate (*n* = 3 to 10 different isolates per condition, 1 to 3 technical replicates per isolate). Mean ± SEM. Differences are assessed by ANOVA (F(10, 61) = 51.51, *P* < 0.0001) and Tukey’s post-hoc tests (*P < 0.05, ***P < 0.0005, ****P < 0.0001). **b** Reticulocyte invasion by parasites with 1 or 3 copies of *pvdbp* in presence of 500 μg ml^−1^ of humabs. Each dot represents the average inhibition of a single clinical isolate (*n* = 3 to 7). Mean ± SEM. Differences are assessed by ANOVA (F(7, 48) = 47.36, *P* < 0.0001) and Tukey’s post-hoc tests (*P < 0.05, **P < 0.01, ***P < 0.0005, ****P < 0.0001). **c** Correlation between the invasion of parasites in presence of 100 μg ml^−1^ of 099100 (black), 053054 (blue) and 092096 (orange) and their level of *pvdbp* mRNA. Each dot represents the mRNA levels and average inhibition of a single clinical isolate (1 to 3 technical replicates per isolate). The correlation is significant for the response to the 092096 humab (Spearman *r* = 0.6703, *P* = 0.0107, the best-fit semi-log correlation line and its 95% confidence interval are represented). **d** Example of dose-response curves obtained for one isolate with a single *pvdbp* copy (open circles) and one isolate with three *pvdbp* copies (plain circles) in presence of 25 to 1000 μg ml^−1^ of 092096. The dots represent the average invasion percent (±SEM) measured from three technical replicates for each isolate. **e** IC_50_ values obtained for parasites with one (*n* = 5 isolates) or three (*n* = 3 isolates) *pvdbp* copies and calculated from dose-response curves using 092096 from 25 to 1000 μg ml^−1^. Each dot represent the average IC_50_ of a different clinical isolate (*n* = 1 to 3 technical replicates per isolate). Mean ± SEM. Mann-Whitney *U* = 0, *P < 0.05. Source data are provided as a Source Data file.

We performed in vitro reticulocyte invasion assays to evaluate how *pvdbp* amplification affects the susceptibility of the parasites to invasion inhibitory human monoclonal antibodies (humabs) targeting the binding domain of PvDBP. Those humabs were previously shown to inhibit the binding of multiple DBPII alleles to the Duffy receptor and to inhibit the reticulocyte invasion by Pv isolates from Brazil and Cambodia^28,29^. In an initial screening, we tested the invasion inhibition activity of three different humabs (099100, 053054 and 092096) at a final concentration of 100 μg ml^−1^ against parasites with one, two or three copies of *pvdbp*. Invasion was normalized to media-alone controls and compared to the invasion measured in presence of 100 μg ml^−1^ of the humab isotype control 043038 specific of tetanus toxin C-terminal fragment^28^. Invasion of parasites with one *pvdbp* copy was significantly inhibited by a mean of 36.1%, 41.6% and 52.6% in presence of 099100, 053054 and 092096 respectively compared to the 043038 controls (Tukey’s post-hoc tests, *P* < 0.0001 for each humab) (Fig. 2a). Inhibition was not significantly different between the three humabs against single-copy parasites (Tukey’s post-hoc tests, *P* > 0.1 for all comparisons). Parasites with two copies of *pvdbp* showed lesser inhibition by humabs compared to the 043038 control with mean of 18.9%, 29.6% and 12.8% inhibition by 099100, 053054 and 092096 respectively, although the inhibition was significant only for the 053054 humab (Tukey’s post-hoc tests, *P* = 0.023). Invasion inhibition was not significantly different between the three humabs against parasites with two *pvdbp* copies (Tukey’s post-hoc tests, *P* > 0.7 for all comparisons). The three monoclonal antibodies at 100 μg ml^−1^ failed to inhibit parasites with three *pvdbp* copies compared to the 043038 control (Tukey’s post-hoc tests, *P* > 0.9 for all comparisons). The response of single copy parasites was significantly lower than of parasites with three copies for all three antibodies (Tukey’s post-hoc tests, *P* = 0.0001 for 099100, *P* = 0.0186 for 053054 and *P* < 0.0001 for 092096). For the three humabs, invasion inhibition of parasites with two copies was lower than of single copies, however the difference was significant only for the 092096 humab (Tukey’s post-hoc tests, *P* = 0.0001). Parasites with three *pvdbp* copies were all less inhibited than two-copy parasites though the difference did not reach significance for any of the humabs (Tukey’s post-hoc tests, *P* > 0.3 for all comparisons). Those results show a gene-dosing effect where the higher the gene copy number, the higher the resistance of the parasites to the inhibitory effect of the humabs.

Increasing the concentration of anti-PvDBP humabs and 043038 isotype control to 500 μg ml^−1^ increased the invasion inhibition against single *pvdbp* copy parasites to 55.1%, 51.6% and 68.3% by 099100, 053054 and 092096 respectively (Tukey’s post-hoc tests, *P* < 0.0001 for each humab compared to 043038) (Fig. 2b). Inhibition was not significantly different between the three humabs against single-copy parasites (Tukey’s post-hoc tests, *P* > 0.3 for all comparisons). When humabs concentration increased to 500 μg ml^−1^, invasion of parasites with three copies of *pvdbp* was inhibited by 33.8%, 38.7% and 28.4% in presence of 099100, 053054 and 092096 respectively. Invasion inhibition was significant for humabs 099100 and 053054 compared to the 043038 control (Tukey’s post-hoc tests, *P* = 0.0233 and *P* = 0.0036 respectively) but not for the 092096 humab (*P* = 0.1362). Again, inhibition was not significantly different between the three humabs against parasites with three copies of *pvdbp* (Tukey’s post-hoc tests, *P* > 0.9 for all comparisons). Inhibition of single copy parasites was higher than of parasites with three *pvdbp* copies for all humabs however the difference was significant only for the 092096 antibody (Tukey’s post-hoc tests, *P* = 0.0007).

By quantifying the level of PvDBP mRNA transcribed in most of the schizonts used for the invasion assays and analyzing the pooled data for all three humabs tested at 100 μg ml^−1^, we could show that invasion in presence of humabs was significantly correlated to the level of mRNA produced (Spearman, r = 0.4226, *P* = 0.0128), consistent with a gene-dosing effect (Fig. 2c). When analyzing per antibody, the correlation was significant only for the 092096 antibody (Spearman, r = 0.6703, *P* = 0.0107) for which there was the highest number of data points (*n* = 14 versus *n* = 9 and *n* = 11 for 053054 and 99100 respectively) suggesting that for the other humabs, the analysis might have been underpowered to detect a correlation.

We performed dose-escalating invasion assays using the 092096 humab from 25 to 1000 μg ml^−1^ against parasites with one or three copies of *pvdbp* allowing to estimate the 50% inhibitory concentrations (IC_50_) (Fig. 2d). On average, parasites with one *pvdbp* copy had an IC_50_ of 140 μg ml^−1^ (±44 SEM) much lower than the mean IC_50_ of 3500 μg ml^−1^ (±2900 SEM) estimated for three *pvdbp* copies parasites (Mann-Whitney, *P* = 0.0357) (Fig. 2e).

Altogether, those data show that increased *pvdbp* copy number leads to in vitro protection of the parasites against the invasion inhibitory activities of anti-PvDBP antibodies.

### Variation in inhibition is independent of PvDBP sequence

We evaluated how the PvDBP sequence polymorphism of the isolates used in the invasion assays affected the response of the parasites to the humabs in order to assess if antigenic variation was responsible for the variations in inhibition observed. Among the 29 isolates used for the in vitro invasion assays, we observed 20 different alleles of DBPII none of which being the Sal1 reference allele, reflecting the known diversity of this gene (Supplementary Table 1)^23^. *pvdbp* amplification was observed in six of those alleles confirming that this gene amplification is not restricted to a particular parasite genotype (Supplementary Fig. 2)^23^.

**Fig. 3:**
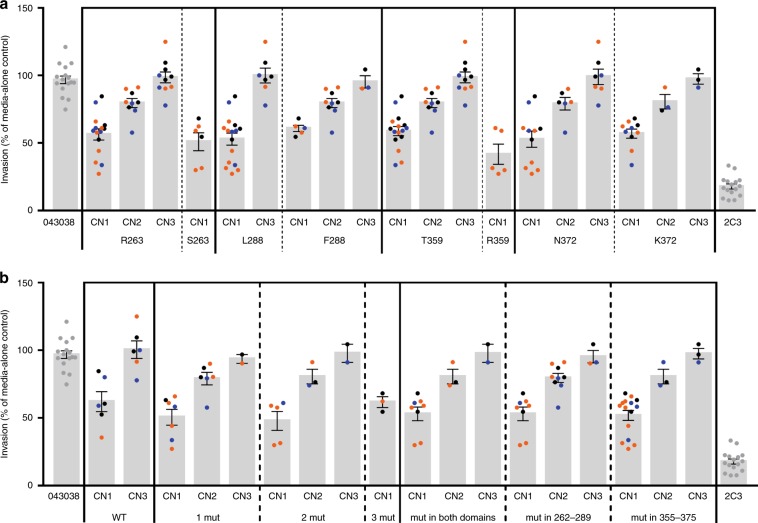
Variation in reticulocyte invasion inhibition by human antibodies is not dependent on the DBPII sequence polymorphism of Pv isolates. Invasion percent of parasites with *pvdbp* copy number of 1 (CN1), 2 (CN2) or 3(CN3) in presence of 100 μg ml^−1^ of 099100 (black), 053054 (blue) or 092096 (orange) human antibodies, 043038 and 2C3 controls. Each dot represents the average invasion measured for a different isolate with 1 to 3 technical replicates per isolate. Mean ± SEM. *P*-values for all significant differences are reported in Supplementary Table 2. **a** Pooled reticulocyte invasion percent by parasites differing by their amino acids at position 263, 288, 359 and 372 within the epitopes of the humabs. For each residue, the wild type (Sal1) amino acid is indicated first followed by the mutant one. **b** Pooled reticulocytes’ invasion percent by parasites either without any mutation in the humab epitopes (WT) or with one (1 mut), two (2 mut), three (3 mut) mutations anywhere within the epitopes, or with any mutation in both domains or either domain 262–289 or 355–375 of the epitopes. Source data are provided as a Source Data file.

We stratified the invasion data by assessing the impact of the polymorphism of DBPII within the epitopes recognized by the humabs. Those epitopes were previously identified by crystallography for 053054 and 092096 and competition experiments have shown that 099100 shared the same or overlapping epitopes^28,29^. The epitopes are made of two domains of the protein, residues 262–289 and 355–375. Of the 29 isolates, eight are wild types in those epitopes and have the same sequence than the Sal1 reference protein used for the isolation of the humabs, while 21 display at least one mutation in any of the two domains of the epitopes (9 parasites with 1 mutation, 9 with 2 mutations and 3 isolates with 3 mutations). For each copy number group, the presence of a SNP at one or more of the four polymorphic residues in the binding domain had no effect on the degree inhibition by anti-DBP monoclonal antibodies compared to the 043038 control (Fig. 3a, b). This lack of association between SNP polymorphisms and blocking activity was the same whether parasites had one, two or three copies of *pvdbp*. In all cases, the only polymorphism driving reduced susceptibility to the humabs is the *pvdbp* copy number.

As most alleles occurred only once among the 29 isolates, we could analyze the effect of *pvdbp* amplification on the response to humabs for a given allele for only two haplotypes. For both haplotypes, variation in invasion inhibition by humabs was driven by the gene copy number though in both cases the number of experiments to analyze were too low to draw significant conclusions (Supplementary Fig. 3).

Altogether, these data confirms that *pvdbp* amplification rather than sequence polymorphism within the humabs epitopes allows the parasites to evade in vitro neutralization by anti-PvDBP monoclonal antibodies.

### Variation in inhibition is independent of Duffy sequence

**Fig. 4:**
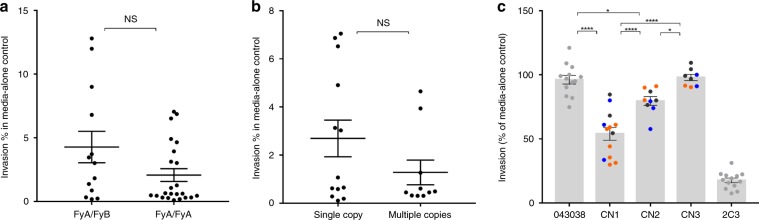
Variation in reticulocyte invasion inhibition by human antibodies is not dependent on the polymorphism of the Duffy receptor of reticulocytes. **a** Similar invasion percent of parasites in FyA/FyB and FyA/FyA reticulocytes in absence of antibodies (NS: non-significant, Mann-Whitney *U* = 108, *P* = 0.1794). Each dot represents the invasion percent of a different clinical isolate with 1 to 3 technical replicates per isolate. Mean ± SEM. **b** Similar invasion percent in FyA/FyA reticulocytes between single and multi copies *pvdbp* parasites in absence of antibodies (Mann-Whitney *U* = 47, *P* = 0.2768). Each dot represents the invasion percent of a different clinical isolate with 1 to 3 technical replicates per isolate. Mean ± SEM. **c** The *pvdbp* copy number of parasites drives variation in inhibition of invasion in FyA/FyA reticulocytes by human antibodies. Pooled invasion percent data in presence of 100 μg ml^−1^ of humabs (black 099100, blue 053054, orange 092096) of isolates with one copy (CN1), two copies (CN2) or three copies (CN3) of pvdbp. ANOVA F (4, 51) = 93.91, *P* < 0.0001, Tukey’s post-hoc tests, *P < 0.05, ****P < 0.0001. Each dot represents the invasion percent of a different clinical isolate with 1 to 3 technical replicates per isolate. Mean ± SEM. Source data are provided as a Source Data file.

On the reticulocyte side, we determined if the sequence polymorphism of the Duffy gene of the reticulocytes used for invasions was related to the variation in invasion inhibition by humabs as the two major Duffy alleles, FyA and FyB, have different binding affinity to PvDBP (FyA having 41–50% lower binding compared to FyB cells)^30^. As expected for South East Asian donors, all genotyped reticulocytes were Duffy positive and the majority of those (16/22) were homozygous FyA while the remaining ones (6/22) were heterozygous FyA/FyB^31^. No other polymorphism was observed among the Duffy sequences of the reticulocytes. In the media-alone controls, the mean invasion percent in FyA/FyB reticulocytes (4.28% ± 1.24 SEM) was higher than in the FyA/FyA ones (2.08% ± 0.49 SEM), however the difference was not significant (Mann-Whitney, *P* = 0.179) (Fig. 4a). When restricting the analysis to FyA/FyA reticulocytes for which there is enough data to assess differences, in absence of antibodies, there was no significant difference in the invasion percent of parasites with single (2.69% ± 0.76 SEM) or multiple copies of *pvdbp* (1.28% ± 0.51 SEM, Mann-Whitney, *P* = 0.277) suggesting that the gene amplification does not promote better invasion (Fig. 4b). In those reticulocytes, pooled invasion data by antibodies at 100 μg ml^−1^ show that single *pvdbp* copy and two copies parasites were significantly inhibited by humabs (Tukey’s post-hoc tests, *P* < 0.0001 and *P* = 0.019 respectively) while invasion of three-copy parasites was not different from invasion in presence of the 043038 control (Tukey’s post-hoc tests, *P* = 0.998, Fig. 4c). In FyA/FyA reticulocytes, invasion of single-copy parasites in presence of humabs was significantly lower than for two copies (Tukey’s post-hoc tests, *P* < 0.0001) and three copies (Tukey’s post-hoc tests, *P* < 0.0001) isolates and two-copy parasites were also significantly more inhibited than three-copy ones (Tukey’s post-hoc tests, *P* = 0.022).

Those results indicate that when controlling for Duffy polymorphism (here restricting to FyA/FyA reticulocytes), the *pvdbp* copy number drives the response of parasites to the humabs.

### *pvdbp* amplification protects Pv in vivo

**Fig. 5:**
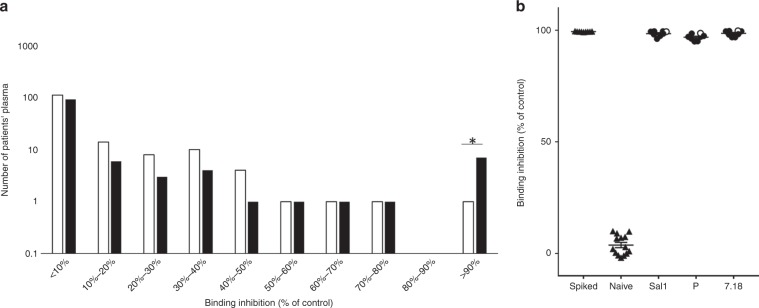
Most Pv parasites infecting individuals displaying antibodies highly inhibiting the PvDBP-red blood cell binding have multiple copies of *pvdbp*. **a** Distribution of parasites with single *pvdbp* copy (*n* = 151, open bars) or multiple copies (*n* = 116, plain bars) across patients with binding inhibitory antibodies ranging from < 10% inhibition to > 90%. Individual plasma (at 1:5 dilution) are tested for their capacity to inhibit the binding of rDBPII (Sal1 allele) to red blood cells. The proportion of *pvdbp*-amplified isolates compared to single copy ones is significantly different only for patients displaying more than 90% binding inhibitory activity (7/116 and 1/151 respectively, Fisher’s exact test, *P* = 0.023). **b** Binding inhibitory antibodies from infected patients are strain transcending. The plasma (at 1:5 dilution) from all eight patients (plain circles infected with multiple *pvdbp* copies parasites and open circles with single copy) show >90% binding inhibition to three different DBPII alleles (Sal1, P and 7.18) similarly to plasma from malaria naïve individuals spiked with the humab 092096. Naïve plasma display <10% binding inhibition. Source data are provided as a Source Data file.

Finally, we sought to determine how the in vitro results would translate in vivo. The parasitemia in patients infected with single-copy parasites (mean parasitemia 8200 parasites per μL ± 726 SEM) was not significantly different than the parasitemia in patients infected with multi-copy parasites (mean 8500 parasites per μL ± 1045 SEM, Mann-Whitney, *P* = 0.9317), which is in line with a previous study^22^. In addition, the proportion of parasites with *pvdbp* amplification was not significantly different between asymptomatic individuals (35/68, 51.5%) and symptomatic ones (101/231, 43.7%, Fisher’s exact test, *P* = 0.2708). We hypothesized that gene amplification could protect parasites in vivo from PvDBP binding inhibitory antibodies (BIAbs). Thus, individuals that presented with blood-stage Pv and had naturally acquired BIAbs would more likely be infected with Pv strains carrying multiple copies of *pvdbp*. Using a recombinant DBPII (rDBPII)-red blood cell binding assay, we screened the plasma of 267 individuals infected with blood-stage Pv, of which 151 (57%) were infected by single copy parasites and 116 (43%) by parasites with multiple *pvdbp* copies. As expected, because BIAbs are both rarely acquired under natural exposure and protective against Pv malaria^16,20^, only 8 individuals had BIAbs highly inhibiting rDBPII-red blood cell binding ( > 90% inhibition at 1:5 plasma dilution) (Fig. 5a). Among those, the proportion of individuals was not significantly different between asymptomatic (4/68, 5.9%) and symptomatic patients (4/199, 2.0%, Fisher’s exact test, *P* = 0.1171). All binding inhibitory plasma were strain transcending and blocked equally the binding of three rDBPII alleles (Sal1, P and 7.18) to erythrocytes (Fig. 5b). Those plasma blocked the rDBPII-red blood cell binding similarly to controls made of naïve plasma spiked with the humab 092096 used as positive inhibitor control. The proportion of patients having plasma with high levels of binding inhibition activity (>90% inhibition at 1:5 plasma dilution) was almost 10 fold higher for patients infected with multiple *pvdbp* copies isolates (7/116, 6.03%) compared to patients infected with single copy parasites (1/151, 0.66%, Fisher’s exact test, *P* = 0.023). Despite the small number of samples, those results seem to indicate that *pvdbp* amplification allows parasites to resist host anti-PvDBP humoral immunity.

## Discussion

The results of this work show that amplification of *pvdbp* allows the parasite to evade host humoral anti-PvDBP immunity. Although this study shows a clear genotype-phenotype association, the underlying molecular mechanisms remain to be elucidated.

Noteworthy, in the work presented here, the levels of inhibition achieved by anti-PvDBP antibodies are significantly lower than the inhibition achieved by blocking the Duffy receptor, except for single-copy parasites in presence of 092096 at 500 μg ml^−1^ (Tukey’s post-hoc tests, *P* = 0.293). This difference could result either (i) from the duration of exposure of merozoite PvDBP to antibodies not long enough to allow complete neutralization therefore requiring very high antibody concentrations to achieve complete inhibition, or (ii) from physical constraints preventing antibodies to fully saturate the parasites’ proteins. If the latter is correct, future studies performing invasion assays using Fab fragments of anti-PvDBP monoclonal antibodies should increase invasion inhibition.

Gene amplification in *Plasmodium* is well described mainly as a mechanism of drug resistance^32,33^ but to our knowledge, it has never been demonstrated as an immune evasion mechanism. Previous studies have shown that a small number of individuals develop high titer BIAbs against DBPII leading to protection against Pv infection^16,20^. These observations supported the development of a vaccine for Pv based on DBPII. It is still not well understood why so few people develop such strain-transcending BIAbs although repeated exposure and perhaps high parasitemia seem to be necessary for their induction^16,18^. The work presented here has implications for a PvDBP vaccine strategy. Pv parasites carrying multiple copies of *pvdbp* are already widespread in many Pv endemic populations and are likely to have evolved to counter high levels of anti-DBPII BIAbs acquired by some individuals. The observation that only 6 to 15% of Pv exposed individuals acquired high levels of strain-transcending anti-DBPII BIAbs^16,20,28^ from several Pv endemic populations may have limited the spread of parasites with multiple *pvdbp* copies.

The results of this work raise the concern that implementation of a DBPII vaccine targeting protective binding epitopes may select for parasites with multiple copies of *pvdbp* thereby reducing its efficacy. Future studies that examine the protective potential of DBPII vaccines in vitro and in vivo need to monitor whether parasites have multiple *pvdbp* copies. Invasion studies with vaccine elicited anti-DBPII antibodies may be used to define binding inhibitory titers that are needed to block invasion of multi-copy *pvdbp* expressing parasites and provide protection against Pv malaria. DBPII-based vaccines may need to be designed such that they can elicit such antibody levels and affinity to mitigate the effect of *pvdbp* amplification. Additionally, a multi-component vaccine associating DBPII and other ligands critical for Pv invasion (such as PvRBP2b^34^) may allow to neutralize multi-copy *pvdbp* parasites.

## Methods

### Sample collection

Pv samples were collected between 2017 and 2019 by active and passive case detection from individuals residing in Mondolkiri, Eastern Cambodia. The presence of Pv was determined using RDT (CareStart^TM^ Malaria Pf/pan RDTs, Accesbio) or microscopy and monoinfections were confirmed by qPCR using species-specific primers^35^. Venous blood used for in vitro invasion assays was collected in lithium heparin tubes and immediately processed on-site in a mobile laboratory. Leukocytes were depleted using NWF filters^36^. The leukocyte-depleted parasitized red blood cells were cryopreserved using glycerolyte 57 solution (Baxter) and immediately stored in liquid nitrogen^37^. Plasma used for the rDBPII-erythrocyte binding assays was collected in EDTA tubes, immediately separated from red blood cells and stored at −80 °C. Ethics clearance for the samples used in this study was obtained from the Ministry of Health National Ethics Committee in Cambodia (317NECHR, 262NECHR) and Institut Pasteur in Paris (2018-10/IRB/1). All patients or their parents/guardians provided informed written consent.

### Gene copy number and sequence polymorphism

The *pvdbp* copy number was determined by qPCR using the tubulin gene as single-copy reference^23^. For low-parasitemia infections, the gene copy number could not be evaluated quantitatively and *pvdbp* amplification was determined using a semi-nested PCR targeting the boundaries of the duplication^22^. We used previously-published primers AR2 and BR^22^ and newly designed primers AF2’ and BF’ (primers listed in Supplementary Table 3) to perform a primary PCR reaction (Supplementary Fig. 4). AR2 + AF2’ are expected to amplify a 683-bp in isolates with or without the *pvdbp* duplication and were used as positive controls. BR + BF’ are expected to amplify a 676-bp region surrounding the duplication boundaries and constitute a positive control as well. BF + AR2 are expected to amplify a 736-bp product containing the junction between the *pvdbp* copies, they are not supposed to amplify DNA in samples without duplication since they are opposite-facing. All primary PCR reactions were carried out with 2 μL of pure DNA, 4 μL of 5x HOT FIREPol Blend Master Mix (Solis Biodyne), 0.5 μL of 10 μM for each reverse and forward primer. Reactions consisted of an initial denaturation of 15 min at 94 °C followed by 25 cycles of 30 s at 94 °C, 30 s at 55 °C, 60 seconds at 72 °C and a final 10 min at 72 °C. For the nested PCR, 2 μL of amplicon were used as template following conditions described in ref. ^22^ using AR2 + AF2 (expected size 657 bp), BR + BF (643 bp) and AR2 + BF (736 bp). Samples with amplification for both controls (AR2 + AF2 and BF + BR) were valid for analysis. If parasites carried the *pvdbp* duplication, a band of 736 bp was observed following PCR using AR2 + BF. In absence of amplification using AR2 + BF, the parasite contains a single *pvdbp* copy^22^.

DBPII sequence polymorphism was determined by Sanger sequencing (Macrogen, Seoul, South Korea)^23^. The Duffy genotypes of the reticulocytes used for the assays were determined by Sanger sequencing (Macrogen, Seoul, South Korea)^24^. Primers are listed in Supplementary Table 3.

### In vitro invasion inhibition assay

Cryopreserved infected RBCs were thawed and cultured in IMDM medium (Gibco) supplemented with 0.5% Albumax II (Gibco), 2.5% heat-inactivated human serum, 25 mM HEPES (Gibco), 20 μg ml^−1^ gentamicin (Sigma) and 0.2 mM hypoxanthine (C-C Pro) for ~24 or ~48 h until a majority of schizont stage parasites were observed. The schizont-infected erythrocytes were enriched using KCl-Percoll density gradient^38^, then mixed at a ratio of 1 erythrocyte to 1 reticulocyte enriched from cord blood and labeled with CellTrace Far Red dye following manufacturer’s instructions. The cultures were incubated for ~8 h in a final volume of 50 μL in 96 well plates or 20 μL in 384 well plates, in presence of the human monoclonal antibodies. Medium alone was used as control for invasion normalization and the mouse monoclonal anti-Duffy 2C3 at 100 μg ml^−1^ was used as positive invasion inhibition controls^37^. Cells were stained with DNA stain Hoechst 33342 post invasion and examined by flow cytometry. Reticulocytes which were Hoechst 33342 and Far Red positive were scored as new invasion events (Supplementary Fig. 5). Invasion of reticulocytes in absence of antibodies ranged from 0.11% to 12.80% (mean 3.2%) for the 41 experiments performed using 29 isolates.

### PvDBP RNA quantification

Following in vitro maturation and KCl-Percoll enrichment as described above, an aliquot of ~15,000 mature schizonts (quantified by flow cytometry or by microscopy following Giemsa staining) was stored in Trizol® LS Reagent (Life Technologies) and RNA was purified using RNeasy Mini Kit from QIAGEN according to the manufacturer’s instruction. RNA was treated with DNase I (Roche) according to the manufacturer’s instruction. PvDBP gene expression was determined in triplicate and normalized to *pvmsp1*, also expressed at the schizont stage. The quantitative real time-PCR was carried out in a final volume of 20 μL using One-Step SuperScript® III RT/Platinum® Taq Mix (Invitrogen). Reactions were carried out with 2 μL of RNA diluted ten times, 10 μL of 2X Reaction Mix, 1 μL of 10 μM reverse and forward primer, and 0.3 μL (PvMSP1) or 0.5 μL (PvDBP) of 10 μM Taqman probe. Reactions consisted of an initial denaturation of 2 min at 95 °C followed by 45 cycles of 15 s at 95 °C, 15 s at 60 °C, and a final 5 min at 35 °C. Primers and probes are listed in Supplementary Table 3.

### PvDBP-red blood cell binding assay

To assess ability of plasma to block binding of DBPII to Duffy positive erythrocytes, a venous blood sample from a single donor (homozygous FyA) was collected using EDTA tubes. Leukocytes were depleted using NWF filters^36^. Ten μL of red blood cell pellet was washed twice in PBS, re-suspended in 200 μL PBS-0.2% BSA for 30 min at 37 °C, then washed twice with PBS-0.2% BSA. A total of 1 × 10^6^ erythrocytes were re-suspended to 50 μL. Plasma (final dilution 1 in 5) was incubated with Sal1 recombinant DBPII protein (final concentration of 0.8 μg ml^−1^) in 10 μL of PBS-0.2% BSA and incubated for 30 min at 37 °C^28^. The mix was added to 1 × 10^6^ erythrocytes in PBS-0.2%BSA and incubated 30 min at 37 °C. After 2 washes with PBS-0.2%BSA, rabbit polyclonal anti-DBPII^39^ at dilution 1/10,000 in 50 μL PBS-0.2%BSA was added and incubated 30 min at 37 °C. Cells were washed twice and incubated at 37 °C in the dark for 30 min with 40 μL anti-rabbit IgG Alexa-Fluor 488-tagged Ab (Life Technology, cat # A11034), at a final dilution of 1/500 in PBS-0.2% BSA. After two washes in PBS-0.2% BSA and a final wash in PBS, erythrocytes were analyzed by flow cytometry. Negative binding inhibitor control was made of malaria-naïve European plasma samples while positive inhibitor control was made of naive plasma spiked with the 092096 humab at 2 μg ml^−1^ final concentration. All bindings were normalized to a no-plasma control. Data were acquired by CyView software and analyzed using FlowJo software v.10 (Tree Star, Inc). The screening of the 267 patients’ plasma was done using Sal1 allele. All inhibitory plasma were then tested against the three rDBPII alleles Sal1, 9 and 7.18 all of which in triplicate experiments.

### Statistical analyses

Comparison of mean of normal data was done by two-sided unpaired t test and of non Gaussian data by two-sided Mann-Whitney’s test. Multiple comparison of mean of normal data were compared by ANOVA and Tukey’s post-hoc tests while non Gaussian data were compared by Kruskal-Wallis test and Dunn’s post-hoc tests. Correlation of non Gaussian variables was assessed by two-tailed Spearman correlation test. Proportions were compared by Fisher’s exact test. All analyses were performed using GraphPad Prism (v7.00).

### Reporting summary

Further information on research design is available in the Nature Research Reporting Summary linked to this article.

## Supplementary information

Supplementary information

Reporting Summary

## Data Availability

The data that support the findings of this study are available from the corresponding authors upon reasonable request. Pv isolates collected from patients cannot be propagated in vitro (no continuous culture) therefore samples are limited in terms of availability. The source data underlying Figs. 1, 2a–e, 3a, b, 4a–c, 5a, b and Supplementary Figs. 1a, b, 2 and 3 are provided as a Source Data file.

## Source Data

Source Data
