# Supplementary material for: Amplification of Duffy binding protein-encoding gene allows Plasmodium vivax to evade host anti-DBP humoral immunity
Source: Nat Commun. 2020 Feb 19;11:953. doi: 10.1038/s41467-020-14574-9 (PMC7031336; doi:10.1038/s41467-020-14574-9)
Supplement: Supplementary file 1 — Supplementary information [file 41467_2020_14574_MOESM1_ESM.pdf]

Supplementary Information

Amplification of Duffy Binding Protein-encoding gene allows *Plasmodium vivax* to evade host anti-DBP humoral immunity

Popovici et al.

Supplementary Table 1. List of SNP and alleles observed in DBPII sequences in isolates used in this work.

In yellow are highlighted the residues lying within the epitopes recognized by the humabs 053054 and 092096[1]. The Sal1 sequence (PVX_110810) was used as reference.

Codon	215	261	263	288	322	326	330	333	339	340	341	345	353	359	372	379	392	405	430	441
Ref nucl	AAA	TTT	AGG	CTT	ATC	AAA	AAT	CGC	GAT	GAA	AAG	CGT	TCT	ACA	AAT	TTA	TGG	CCC	CCA	CAA
Ref AA	K	F	R	L	I	K	N	D	D	E	K	R	S	T	N	L	W	P	P	Q
Mut nucl	GAA	TTG	AGT	TTT	ACC	GAA	GAT	CGT	GGT	AAA	AAT/CAG	CAT	ACT	AGA	AAA	ATA	CGG	CGC	GCA	GAA
Mut AA	E	L	S	F	T	E	D	G	G	K	N/Q	H	T	R	K	I	R	R	A	E
**allele number**																				
1																		R		
2									G			H								
3									G			H								E
4					T				G			H				I				
5	E														K	I	R			
6				F												I	R		A	
7				F												I	R		A	
8				F					G			H				I	R			
9						E			G		Q				K	I	R			
10				F					G			H			K	I	R			
11	E					E			G		Q				K	I	R			
12				F		E			G	K	N				K	I	R			
13									G	K	N	H	T	R		I				E
14		L	S						G	K	N	H	T	R		I				
15			S						G	K	N	H	T	R		I				E
16			S	F			D		G	K	N	H			K	I	R			
17		L							G	K	N	H	T	R		I				
18	E																	R		E
19		L	S					G	G	K	N	H	T	R		I				
20			S	F			D	G	G	K	N	H			K	I	R			

Supplementary Table 2. P values for all significant comparisons (P<0.05) of invasion data stratified by the sequence polymorphism in PvDBP of parasites described in Figure 3a and 3b.

Fig 3a	P Value	Fig 3b	P Value
**Position 263: ANOVA F (5, 66) = 89.32 P<0.0001**		**WT, 1, 2 or 3 mutations: Kruskal-Wallis H=61.29, P<0.0001**	
**Tukey's multiple comparisons test**		**Dunn's multiple comparisons test**	
WT R263_1 copy vs. WT R263_2 copies	0.0004	043038 vs. 1 mutation_1 copy	0.0466
WT R263_1 copy vs. WT R263_3 copies	<0.0001	043038 vs. 2C3	<0.0001
WT R263_1 copy vs. 043038	<0.0001	WT_3 copies vs. 2C3	<0.0001
WT R263_1 copy vs. 2C3	<0.0001	**WT, mutations in both domains: Kriskal-Wallis H= 47.9, P<0.0001**	
WT R263_2 copies vs. WT R263_3 copies	0.0153	**Dunn's multiple comparisons test**	
WT R263_2 copies vs. mut S263_1 copy	0.0005	043038 vs. 2C3	<0.0001
WT R263_2 copies vs. 043038	0.0158	WT_3 copies vs. 2C3	<0.0001
WT R263_2 copies vs. 2C3	<0.0001	**WT, mutation in domain 262-289: Kruskal-Wallis, H= 53.44, P<0.0001**	
WT R263_3 copies vs. mut S263_1 copy	<0.0001	**Dunn's multiple comparisons test**	
WT R263_3 copies vs. 2C3	<0.0001	043038 vs. 262-289_1 copy	0.014
mut S263_1 copy vs. 043038	<0.0001	043038 vs. 2C3	<0.0001
mut S263_1 copy vs. 2C3	<0.0001	WT_3 copies vs. 2C3	<0.0001
043038 vs. 2C3	<0.0001	262-289_2 copies vs. 2C3	0.0115
**Position 288: ANOVA F (6, 65) = 74.66, P<0.0001**		262-289_3 copies vs. 2C3	0.0121
**Tukey's multiple comparisons test**		**WT, mutations in domain 355-375, Kruskal-Wallis H=55.47, P<0.0001**	
WT L288_1 copy vs. WT L288_3 copies	<0.0001	**Dunn's multiple comparisons test**	
WT L288_1 copy vs. mut F288_2 copies	<0.0001	043038 vs. 355-375_1 copy	0.003
WT L288_1 copy vs. mut F288_3 copies	<0.0001	043038 vs. 2C3	<0.0001
WT L288_1 copy vs. 043038	<0.0001	WT_3 copies vs. 355-375_1 copy	0.0358
WT L288_1 copy vs. 2C3	<0.0001	WT_3 copies vs. 2C3	<0.0001
WT L288_3 copies vs. mut F288_1 copy	<0.0001	355-375_3 copies vs. 2C3	0.0056
WT L288_3 copies vs. mut F288_2 copies	0.025		
WT L288_3 copies vs. 2C3	<0.0001		
mut F288_1 copy vs. mut F288_3 copies	0.0047		
mut F288_1 copy vs. 043038	<0.0001		
mut F288_1 copy vs. 2C3	<0.0001		
mut F288_2 copies vs. 043038	0.0211		
mut F288_2 copies vs. 2C3	<0.0001		
mut F288_3 copies vs. 2C3	<0.0001		
043038 vs. 2C3	<0.0001		
**Position 359: ANOVA F (5, 66) = 100.7, P<0.0001**			
**Tukey's multiple comparisons test**			
WT T359_1 copy vs. WT T359_2 copies	0.0009		
WT T359_1 copy vs. WT T359_3 copies	<0.0001		

WT T359_1 copy vs. 043038	<0.0001
WT T359_1 copy vs. 2C3	<0.0001
WT T359_2 copies vs. WT T359_3 copies	0.0089
WT T359_2 copies vs. mut R359_1 copy	<0.0001
WT T359_2 copies vs. 043038	0.0092
WT T359_2 copies vs. 2C3	<0.0001
WT T359_3 copies vs. mut R359_1 copy	<0.0001
WT T359_3 copies vs. 2C3	<0.0001
mut R359_1 copy vs. 043038	<0.0001
mut R359_1 copy vs. 2C3	0.0021
043038 vs. 2C3	<0.0001
**Position 372: ANOVA F (7, 64) = 61.68, P<0.0001**	
**Tukey's multiple comparisons test**	
WT N372_1 copy vs. WT N372_2 copies	0.0024
WT N372_1 copy vs. WT N372_3 copies	<0.0001
WT N372_1 copy vs. mut K372_2 copies	0.0231
WT N372_1 copy vs. mut K372_3 copies	<0.0001
WT N372_1 copy vs. 043038	<0.0001
WT N372_1 copy vs. 2C3	<0.0001
WT N372_2 copies vs. mut K372_1 copy	0.0217
WT N372_2 copies vs. 2C3	<0.0001
WT N372_3 copies vs. mut K372_1 copy	<0.0001
WT N372_3 copies vs. mut K372_2 copies	0.3979
WT N372_3 copies vs. 2C3	<0.0001
mut K372_1 copy vs. mut K372_3 copies	0.0002
mut K372_1 copy vs. 043038	<0.0001
mut K372_1 copy vs. 2C3	<0.0001
mut K372_2 copies vs. 2C3	<0.0001
mut K372_3 copies vs. 2C3	<0.0001
043038 vs. 2C3	<0.0001

Supplementary Table 3. Primers and probes used in this work

Name	Sequence	Ref	
RTPCR_2_Pv_DBP_Probe	5'- [6FAM]ATGGGAACGGATATGGAAGGCATCG[BHQ1] -3'	This work	**PvDBP RNA quantification**
RTPCR_2_Pv_DBP_R	5'-GCGCAAATTATTTTCCACTACTTTG-3'	This work	
RTPCR_2_Pv_DBP_F	5'-GCA GGATATAAGATGGAGTTTGG-3'	This work	
RTPCR_Pv_MSP1_R	5'-GCATTATCAGGCACATTGGTG-3'	This work	
RTPCR_Pv_MSP1_probe	5'- [6FAM]CTGAGTTTGCACATTTAGCAGCTGGG[BHQ1] -3'	This work	
RTPCR_Pv_MSP1_F	5'-TTGATTAAAGAAAACGAGTCCAAGG-3'	This work	
PCR Fy F	5'-GTGGGGTAAGGCTTCCTGAT-3'	2	**Duffy sequence polymorphism**
PCR Fy R	5'-CAGAGCTGCGAGTGCTACCT-3'	2	
Nested GATA	5'-CAAACAGCAGGGGAAATGAG-3'	2	
Nested Fy SNP	5'-CTTCCGGTGTAACTCTGATGG-3'	2	
CN_PvDBP_F	5′-AATTATAAGAGAAAACGTCGGGAAAG-3′	3	**PvDBP gene copy number quantification**
CN_PvDBP_R	5′-ACCAAATTCGTAAGTTCCTTCATACA -3′	3	
CN_β-tubulin_F	5′-CATGTTCGTTAAGATTTCCTGGT-3′	3	
CN_β-tubulin_R	5′-GTTAGTGGTGCAAAACCAATCA-3′	3	
PvDBP	GAAAAACTGTAATTATAAGAGAAAACGTCGGGAAAGAGATTGGGACTGTAACACTAAGAAGGATGTTTGTATACCAGATCGAAGATATCAATTATGTATGAAGGAACTTACGAATTTGGTAAATAATACA	3	**Synthetic genes for PvDBP gene copy number quantification**
β-TubulinPv	CAGGAGTTACATGTTCGTTAAGATTTCCTGGTCAGTTAAATTCTGATTTGAGAAAATTAGCTGTCAATTTAATTCCCTTCCCAAGACTCCACTTTTTTATGATTGGTTTTGCACCACTAACAAGCAGAGG	3	
AR2	5’-TAGAACGCACAGTTATTGGC-3’	4	**PvDBP duplication boundaries PCR**
AF2	5′-ACGCGATGTATCTTCTTTTCA-3′	4	
AF2’	5′-TCACGTATCCCAGAGTGCA-3	This work	
BR	5’-TTGCACGTACTCGAAACTCAG-3’	4	
BF	5’-TCATCGAGCATGTTCCTTTG-3’	4	
BF’	5’- GGTAACTTACAACCATTGGTC-3’	This work	
PvDBPsd_PF :	5'-GCATGAGGGAAATTCTCGTA-3'	3	**PvDBPII sequence polymorphism**
PvDBPsd_PR:	5'-CGTTAAATTCATCTAACTCCTGTTT-3'	3	
PvDBPsd_NF:	5'-GAATGGTGGCAATCCTTACG-3'	3	
PvDBPsd_NR:	5'-TCTGAACCTTTTCTGCGTTTT-3'	3	

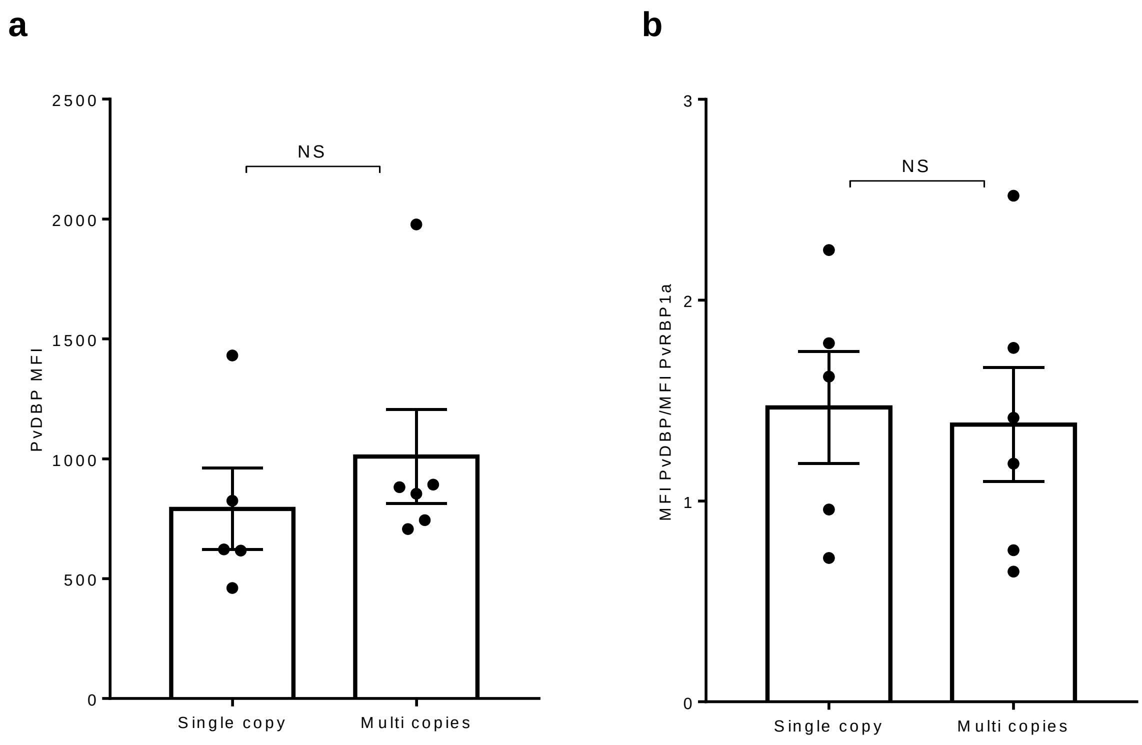

Supplementary Figure 1. PvDBP protein quantification

a. Flow cytometry MFI values obtained from analysis of mature schizonts using rabbit polyclonal anti-PvDBP followed by anti-rabbit Alexa Fluor 488 secondary antibody for single (N=5 isolates) and multi (N=6 isolates) copy parasites (Mann-Whitney, U=7, P=0.1775). **b.** The level of PvRBP1a was quantified using rabbit polyclonal anti-PvRBP1a on the same schizonts and used to normalize DBP MFI values to correct for any difference in schizont maturity between isolates. (Unpaired t test, t=0.2108 df=9, P=0.8378). Means ± SEM are represented in **a** and **b**. Source data are provided as a Source Data file.

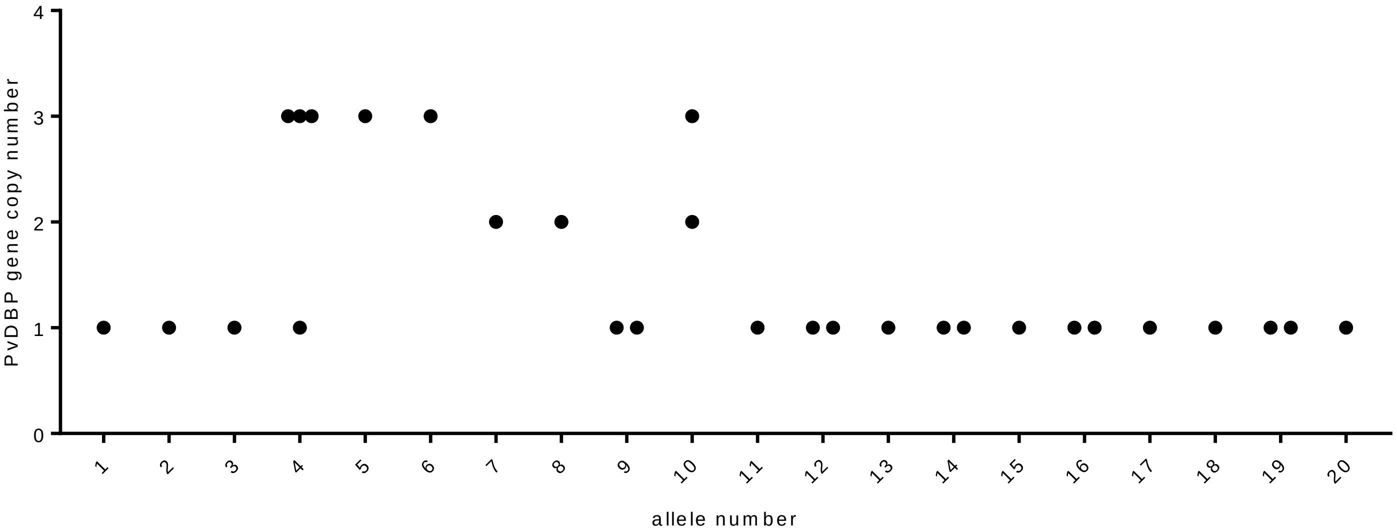

Supplementary Figure 2. Distribution of *pvdbp* copy number across the 20 DBPII alleles among isolates used in this work.

Each dot represents a different isolate. For example, among the parasites with the allele 4, one isolate had a single *pvdbp* copy and three isolates had three copies. Source data are provided as a Source Data file.

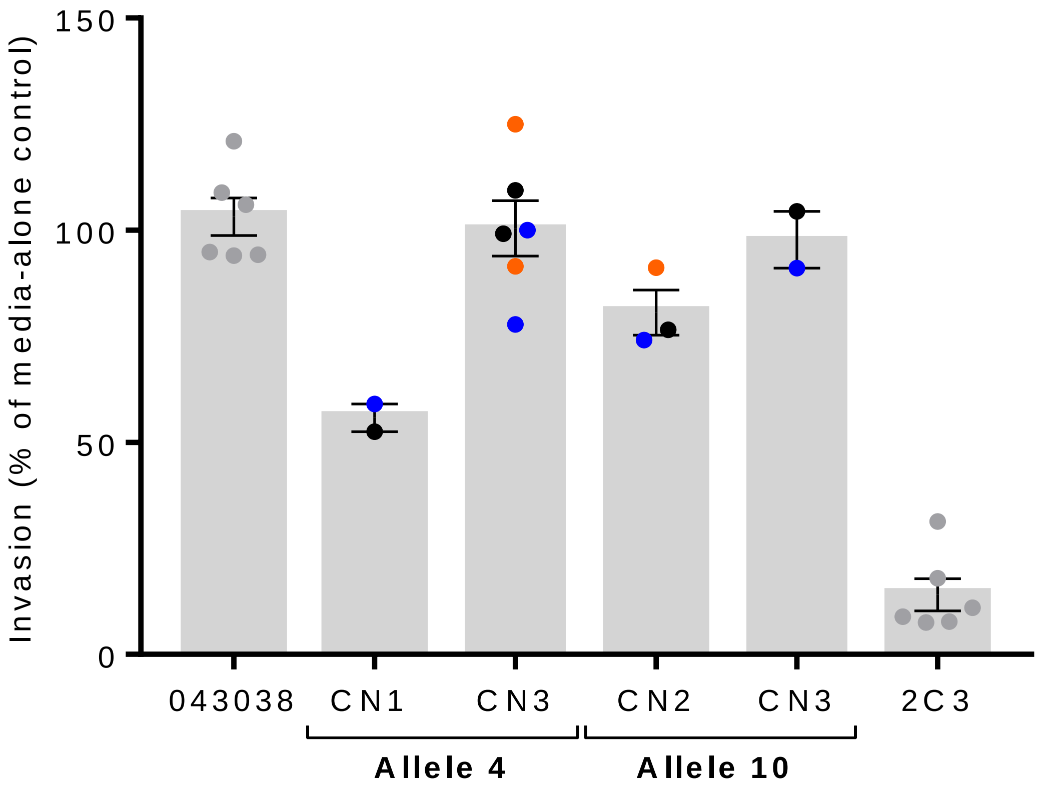

Supplementary Figure 3. Response to humabs of parasites with a same DBPII allele is driven by the gene copy number.

Pooled reticulocyte invasion percent by parasites with a same DBPII allele and a *pvdbp* copy number of 1 (CN1) or 3 (CN3) (for allele 4) or with a copy number of 2 (CN2) or 3 (for allele 10) in presence of 100 µg ml^{-1} of 099100 (black), 053054 (blue) or 092096 (orange). Mean ± SEM are represented. Source data are provided as a Source Data file.

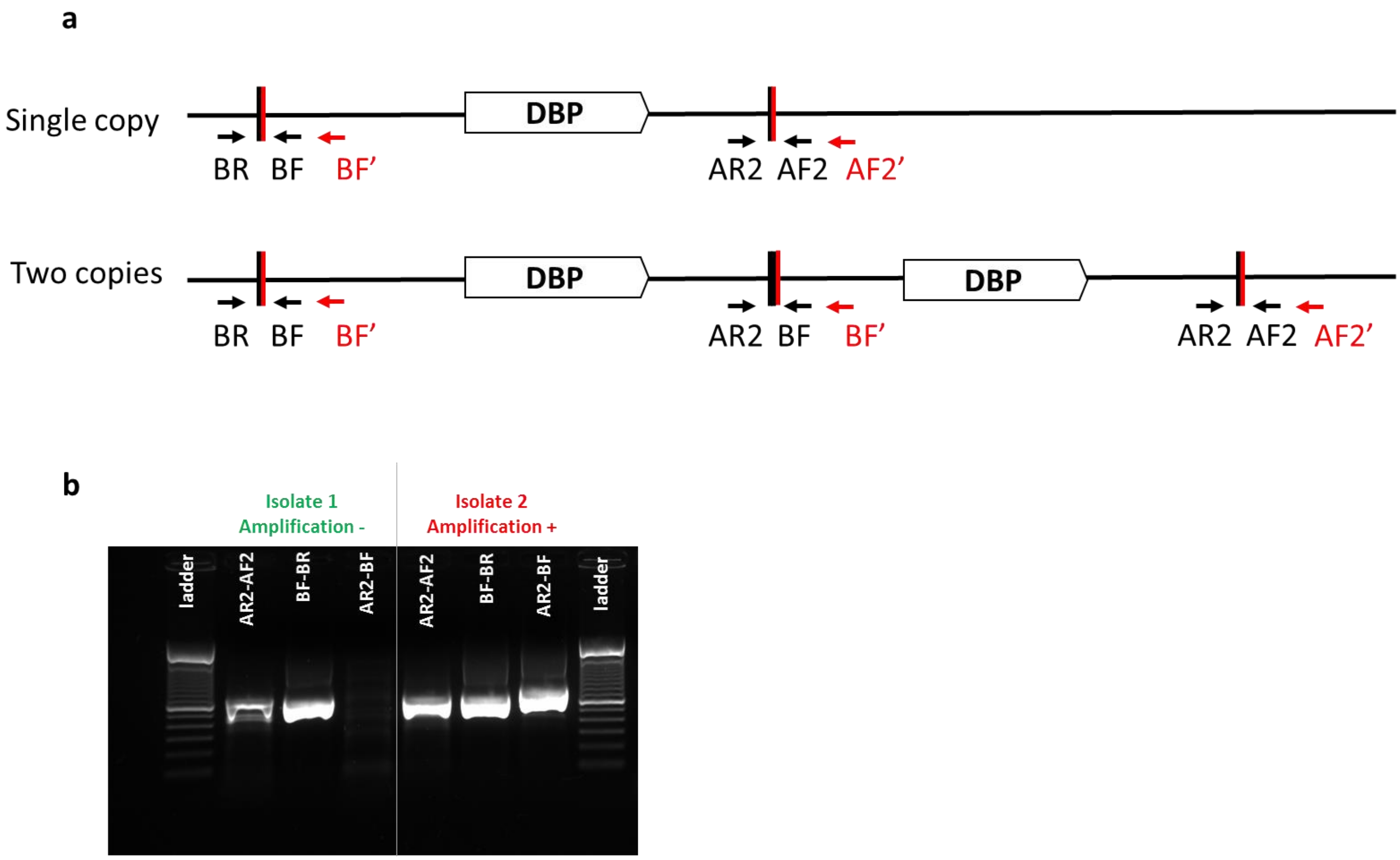

Supplementary Figure 4. PvDBP amplification PCR-based determination

a. Schematic representation of the semi-nested PCR targeting the boundaries of the *pvdbp* duplication adapted from Hostetler et al[4]. Previously-published primers BR, BF, AR2 and AF2 are represented in black and new primers AF2’ and BF’ in red. The primary PCR used the primer pairs BR-BF’, AR2-AF2’ and AR2-BF’ and the secondary PCR used the previously published primer pairs BR-BF, AR2-AF2 and AR2-BF. **b.** Samples are considered valid if a 657bp and a 643bp band are amplified using AR2-AF2 and BF-BR primer pairs respectively. A 736bp band will be amplified using AR2-BF primer pair if the parasite carries the *pvdbp* duplication while no band will be observed for single-*pvdbp* copy parasites.

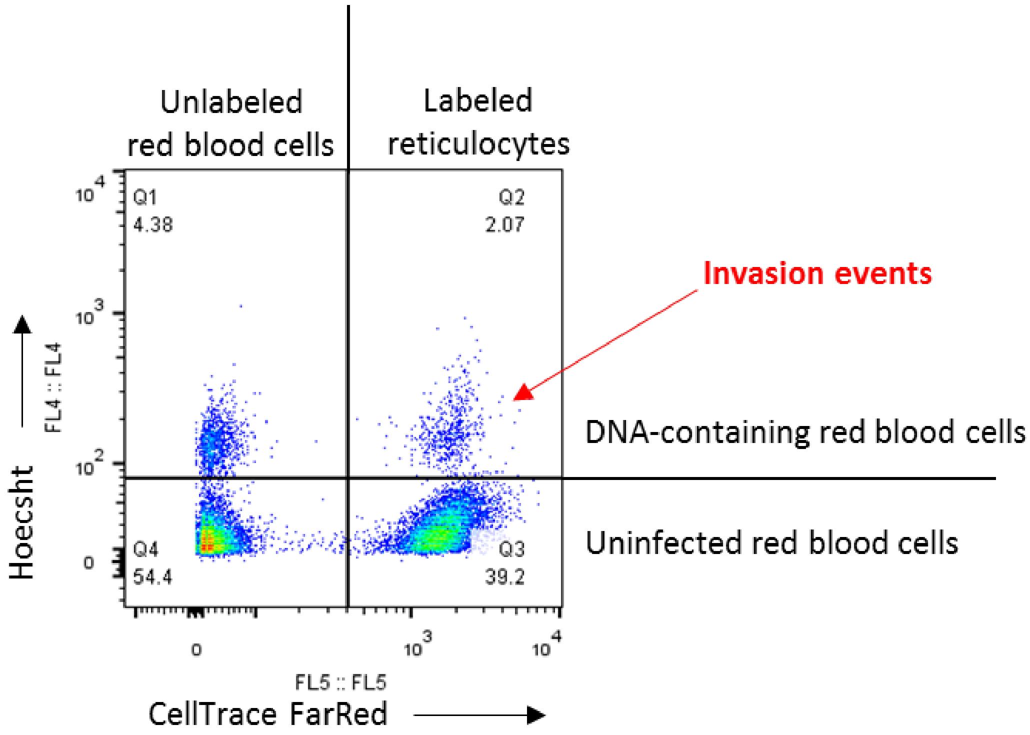

Supplementary Fig 5. Flow cytometry gating and scoring of *P. vivax* invasion in Cell-Trace Far Red labelled reticulocytes following DNA staining with Hoechst dye.

Supplementary methods

PvDBP expression quantification

Percoll enriched schizonts were fixed with paraformaldehyde using the True Nuclear Transcription kit (BioLegend) following the manufacturer's recommendations. Rabbit polyclonal anti-PvDBP or anti-PvRBP1a[5] were diluted in the kit's Perm buffer (1 in 500 dilution), added to fixed schizonts and incubated 30 minutes at 37ºC. Cells were washed twice with PBS-BSA 0.2% then anti-rabbit Alexa Fluor 488 (Life Technology, cat # A11034, diluted at 1 in 500 in PBS-BSA 0.2%) with 20 µg ml^{-1} of Hoechst 33342 dye were added to the cells and incubated 30 minutes at 37ºC. Cells were washed three times in PBS-BSA 0.2% before being analyzed by flow cytometry (gating described below).

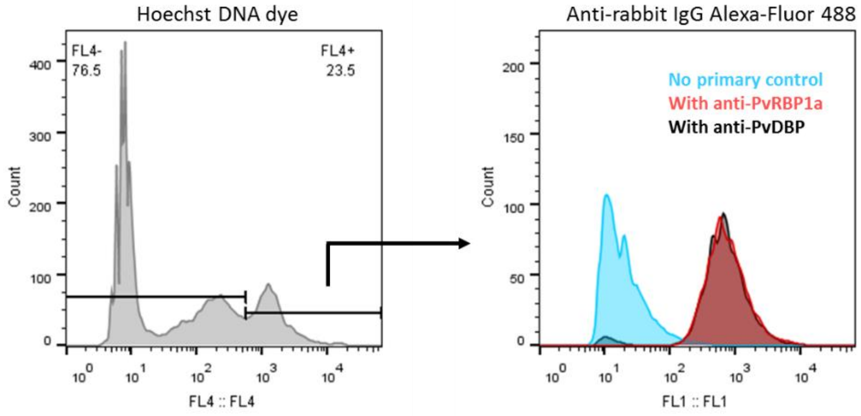

Flow cytometry gating strategy to measure PvDBP and PvRBP1a levels in mature schizonts. Schizonts (with the highest DNA content as containing multiple merozoites) are first gated on FL4 (Hoechst dye) parameter and MFI from anti-rabbit Alexa Fluor 488 signal is measured on FL1 parameter.

Supplementary references

1. Urusova, D., et al. Structural basis for neutralization of Plasmodium vivax by naturally acquired human antibodies that target DBP. *Nat. Microbiol*. **4**, 1486-1496 (2019).

2. Ménard, D., et al. Plasmodium vivax clinical malaria is commonly observed in Duffy-negative Malagasy people. *Proc. Natl. Acad. Sci. U. S. A.* **107**, 5967-5971 (2010).

3. Roesch, C., et al. Genetic diversity in two Plasmodium vivax protein ligands for reticulocyte invasion. *PLOS Neg. Trop. Dis*. **12**, e0006555-e0006555 (2018).

4. Hostetler, J.B., et al. Independent Origin and Global Distribution of Distinct Plasmodium vivax Duffy Binding Protein Gene Duplications. *PLOS Neg. Trop. Dis*. **10**, e0005091 (2016).

5. Gupta, S., et al. Targeting a Reticulocyte Binding Protein and Duffy Binding Protein to Inhibit Reticulocyte Invasion by Plasmodium vivax. *Sci. Rep*. **8**, 10511-10511 (2018)
