# Supplementary material for: Amplification of Duffy binding protein-encoding gene allows Plasmodium vivax to evade host anti-DBP humoral immunity
Source: Nat Commun. 2020 Feb 19;11:953. doi: 10.1038/s41467-020-14574-9 (PMC7031336; doi:10.1038/s41467-020-14574-9)
Supplement: Supplementary file 2 — Reporting Summary [file 41467_2020_14574_MOESM2_ESM.pdf]

nature research

Corresponding author(s): Jean Popovici and Benoit Witkowski

Last updated by author(s): Oct 25, 2019

# Reporting Summary

Nature Research wishes to improve the reproducibility of the work that we publish. This form provides structure for consistency and transparency in reporting. For further information on Nature Research policies, seeAuthors & Referees and theEditorial Policy Checklist .

## Statistics

For all statistical analyses, confirm that the following items are present in the figure legend, table legend, main text, or Methods section.

| n/a | Confirmed | |
|---|---|---|
| ☐ | ☒ | The exact sample size (*n*) for each experimental group/condition, given as a discrete number and unit of measurement |
| ☐ | ☒ | A statement on whether measurements were taken from distinct samples or whether the same sample was measured repeatedly |
| ☐ | ☒ | The statistical test(s) used AND whether they are one- or two-sided<br>*Only common tests should be described solely by name; describe more complex techniques in the Methods section.* |
| ☒ | ☐ | A description of all covariates tested |
| ☐ | ☒ | A description of any assumptions or corrections, such as tests of normality and adjustment for multiple comparisons |
| ☐ | ☒ | A full description of the statistical parameters including central tendency (e.g. means) or other basic estimates (e.g. regression coefficient) AND variation (e.g. standard deviation) or associated estimates of uncertainty (e.g. confidence intervals) |
| ☐ | ☒ | For null hypothesis testing, the test statistic (e.g. *F*, *t*, *r*) with confidence intervals, effect sizes, degrees of freedom and *P* value noted<br>*Give P values as exact values whenever suitable.* |
| ☒ | ☐ | For Bayesian analysis, information on the choice of priors and Markov chain Monte Carlo settings |
| ☒ | ☐ | For hierarchical and complex designs, identification of the appropriate level for tests and full reporting of outcomes |
| ☒ | ☐ | Estimates of effect sizes (e.g. Cohen's *d*, Pearson's *r*), indicating how they were calculated |

*Our web collection on statistics for biologists contains articles on many of the points above.*

## Software and code

Policy information about availability of computer code

| | |
|---|---|
| Data collection | Flow cytometry data were collected using CyView v1.6.4.10 |
| Data analysis | FlowJo v10 |

For manuscripts utilizing custom algorithms or software that are central to the research but not yet described in published literature, software must be made available to editors/reviewers. We strongly encourage code deposition in a community repository (e.g. GitHub). See the Nature Research guidelines for submitting code & software for further information.

## Data

Policy information about availability of data

All manuscripts must include a data availability statement. This statement should provide the following information, where applicable:

- Accession codes, unique identifiers, or web links for publicly available datasets
- A list of figures that have associated raw data
- A description of any restrictions on data availability

The data that support the findings of this study are available from the corresponding authors upon reasonable request P. vivax isolates collected from patients cannot be propagated in vitro (no continuous culture) therefore samples are limited in terms of availability

# Field-specific reporting

Please select the one below that is the best fit for your research. If you are not sure, read the appropriate sections before making your selection.

☒ Life sciences ☐ Behavioural & social sciences ☐ Ecological, evolutionary & environmental sciences

# Life sciences study design

All studies must disclose on these points even when the disclosure is negative.

| | |
|---|---|
| Sample size | No sampe-size calculation was performed. Sample sizes were sufficient to carry out all the required experiments with sufficient statistics and standard for such experiments. |
| Data exclusions | PvDBP gene amplification was not interpretable in very low parasitemia samples where both PCR controls (AR2-AF2 and BR-BF) were not amplified, therefore those were not included in the final analysis. No other data was excluded. The exclusion criteria were pre-established |
| Replication | All data reported were generated using at least three different clinical isolates for each condition (biological replicate) with 1 to 3 technical replicate per clinical isolate for each condition evaluated depending on sample availability. All attempts at replication were successful. |
| Randomization | Randomization is not relevant to the study design mainly based on in vitro data |
| Blinding | Binding inhibition assays were carried out blinded to the gene copy number of the infecting parasites. No other experiment was performed blinded |

# Reporting for specific materials, systems and methods

We require information from authors about some types of materials, experimental systems and methods used in many studies. Here, indicate whether each material, system or method listed is relevant to your study. If you are not sure if a list item applies to your research, read the appropriate section before selecting a response.

## Materials & experimental systems

| n/a | Involved in the study | |
|---|---|---|
| ☐ | ☒ | Antibodies |
| ☒ | ☐ | Eukaryotic cell lines |
| ☒ | ☐ | Palaeontology |
| ☒ | ☐ | Animals and other organisms |
| ☐ | ☒ | Human research participants |
| ☒ | ☐ | Clinical data |

## Methods

| n/a | Involved in the study | |
|---|---|---|
| ☒ | ☐ | ChIP-seq |
| ☐ | ☒ | Flow cytometry |
| ☒ | ☐ | MRI-based neuroimaging |

## Antibodies

| | |
|---|---|
| Antibodies used | Human monoclonal antibodies and rabbit polyclonal antibodies to P. vivax Duffy binding protein and PvRBP1a were characterized in previous studies (Carias et al, 2019, J. Immunol. 202, 2648-2660; Urusova et al 2019, Nat. Microbio.; Gupta et al 2018, Sci. Rep. 8, 10511-10511). The 2C3 mouse primary monoclonal antibody to DARC was characterized in Smolarek et al Cell. Mol. Life Sci. 2010, 67:3371–3387. Goat anti-rabbit Alexa Fluor 488 was purchased from Life Technologies (reference A11034). |
| Validation | The commercially available antibodies can be identified on the manufacturers' websites as indicated above The monoclonal and polyclonal antibodies are described in prior studies (see above). |

## Human research participants

Policy information about studies involving human research participants

| | |
|---|---|
| Population characteristics | Cambodian patients infected with P. vivax parasites, male and female, aged more than 15 years old. |
| Recruitment | Patients were recruited through cross sectional surveys or in malaria clinics and offered participation in the studies (supply of parasite-infected blood sample) after the study aims and protocol have been described. Written informed consent was obtained from all. Participation in the study did not affect treatment practices; malaria treatment was not provided by the research team, but by the clinical staff of the malaria outposts. |
| Ethics oversight | Cambodian National Ethics Committee on Health Research and Institut Pasteur Ethics Committee |

Note that full information on the approval of the study protocol must also be provided in the manuscript.

# Flow Cytometry

## Plots

Confirm that:

- [ ] The axis labels state the marker and fluorochrome used (e.g. CD4-FITC).
- [x] The axis scales are clearly visible. Include numbers along axes only for bottom left plot of group (a 'group' is an analysis of identical markers).
- [x] All plots are contour plots with outliers or pseudocolor plots.
- [x] A numerical value for number of cells or percentage (with statistics) is provided.

## Methodology

**Sample preparation**

Sample preparation is described in the Methods section. For the binding inhibition assays, rDBPII and plasma were preincubated together before addition to RBCs of the rDBPII/plasma mixture. Polyclonal anti-DBPII were then added followed by anti-rabbit Alexa-Fluor 488 secondary antibody. For the invasion studies, deleukocyted cryopreserved iRBCs obtained from Cambodian patients with acute P.vivax malaria were thawed and cultured in IMDM medium. The schizont-infected erythrocytes were enriched using Percoll-KCl, then mixed at a ratio of 1 erythrocyte to 1 with reticulocytes enriched by Percoll from cord blood and labeled with CellTrace Far Red dye (DDAO). Cells were then stained with DNA stain Hoechst 33342. For PvDBP quantification, enriched schizonts were fixed, incubated with either rabbit polyclonal PvDBP or PvRBP1a then incubated with anti-rabbit Alexa-Fluor 488 secondary antibody and Hoecsht 33342 dye.

**Instrument**

Partec Cube 8

**Software**

Data collection: CyView v1.6.4.10 and analysis: FlowJo v10

**Cell population abundance**

No cell sorting was performed.

**Gating strategy**

For the binding inhibition assays, red blood cells (the only cell type) are gated on FSC/SSC then cells are analysed on the FL1 parameter. Boundary between FL1+ (AlexaFluor 488+) and – cells is defined at the bottom of the peak of no-rDBPII controls. For the invasion studies, red blood cells (the only cell type) are gated on FSC/SSC. Quadrant gates are used on the FL4 (Hoescht) FL5 (DDAO) pseudocoulor blots. For the PvDBP quantification, schizonts are gated on the FL4 parameter for DNA content then cells are analysed on the FL1 parameter. Boundary between FL1+ (AlexaFluor 488+) and – cells is defined at the bottom of the peak of no-primary antibody controls.

- [x] Tick this box to confirm that a figure exemplifying the gating strategy is provided in the Supplementary Information.
